# Laparoscopic versus open adhesiolysis for small bowel obstruction - a multicenter, prospective, randomized, controlled trial

**DOI:** 10.1186/1471-2482-14-77

**Published:** 2014-10-11

**Authors:** Ville Sallinen, Heidi Wikström, Mikael Victorzon, Paulina Salminen, Vesa Koivukangas, Eija Haukijärvi, Berndt Enholm, Ari Leppäniemi, Panu Mentula

**Affiliations:** 1 Department of Abdominal Surgery, Helsinki University Central Hospital, Haartmaninkatu 4, 00029 Helsinki, Finland; 2 Vaasa Central Hospital, Hietalahdenkatu 2-4, 65130 Vaasa, Finland; 3 Turku University Hospital, PL 52, 20521 Turku, Finland; 4 Oulu University Hospital, Kajaanintie 50, 90220 Oulu, Finland; 5 Tampere University Hospital, PL 2000, 33521 Tampere, Finland; 6 Päijät-Häme Central Hospital, Keskussairaalankatu 7, 15850 Lahti, Finland

## Abstract

**Background:**

Laparoscopic adhesiolysis is emerging as an alternative for open surgery in adhesive small bowel obstruction. Retrospective studies suggest that laparoscopic approach shortens hospital stay and reduces complications in these patients. However, no prospective, randomized, controlled trials comparing laparoscopy to open surgery have been published.

**Methods/Design:**

This is a multicenter, prospective, open label, randomized, controlled trial comparing laparoscopic adhesiolysis to open surgery in patients with computed-tomography diagnosed adhesive small bowel obstruction that is not resolving with conservative management. The primary study endpoint is the length of postoperative hospital stay in days.

Sample size was estimated based on preliminary retrospective cohort, which suggested that 102 patients would provide 80% power to detect a difference of 2.5 days in the length of postoperative hospital stay with significance level of 0.05. Secondary endpoints include passage of stool, commencement of enteral nutrition, 30-day mortality, complications, postoperative pain, and the length of sick leave. Tertiary endpoints consist of the rate of ventral hernia and the recurrence of small bowel obstruction during long-term follow-up. Long-term follow-up by letter or telephone interview will take place at 1, 5, and 10 years.

**Discussion:**

To the best of our knowledge, this trial is the first one aiming to provide level Ib evidence to assess the use of laparoscopy in the treatment of adhesive small bowel obstruction.

**Trial registration:**

ClinicalTrials.gov identifier: NCT01867528. Date of registration May 26th 2013.

## Background

Small bowel obstruction (SBO) is a common surgical emergency most frequently caused by adhesions. A large portion of adhesive SBO resolve by nonoperative methods such as fasting and ingestion of an oral contrast-media, while a significant number of patients will need emergency surgery [1]. For decades open surgery has been the gold standard in treating adhesive SBO. Now that laparoscopic surgery has been established as a first line option in many elective indications such as colorectal surgery, fundoplication, and cholecystectomy for example, laparoscopy is emerging also as a viable alternative in emergency surgery.

If SBO is caused by one adhesive band, the surgical treatment is straightforward - cutting the band causing obstruction. Laparoscopic approach seems ideal for such a procedure, preventing the morbidity of a laparotomy incision. First publications describing laparoscopic adhesiolysis in SBO are from the 1990’s [2]. Since then several retrospective series have been published, and a recent meta-analysis pooled patients from four studies, including a total of 334 patients [3]. Meta-analysis showed that patients treated by the laparoscopic approach had less complications, and faster return of bowel function [3]. However, there are no prospective randomized trials comparing open approach to laparoscopy. Furthermore, previous retrospective studies have a selection bias because the easiest cases are selected for laparoscopic approach. One of the drawbacks of laparoscopic approach is a concern for iatrogenic bowel perforation. In one report, the rate of bowel lesion in laparoscopic adhesiolysis was 6.6%, and only 84% were detected during the operation [4].

## Methods/Design

### Objective

The objective of this trial is to compare open surgery to laparoscopic adhesiolysis in patients with computed tomography-diagnosed adhesive SBO that is not resolved by nonoperative means. The hypothesis is that laparoscopic approach shortens the length of hospital stay without increasing complications.

### Ethics and permissions

This study will be conducted in accordance with the principles of the Declaration of Helsinki and ‘good clinical practice’ guidelines. The research plan has been evaluated and approved by the local institutional ethics committee of the main research center (Helsinki University Central Hospital, Ethics Committee, Department of Surgery). The research plan has further been approved by each participating centers’ institutional review board (Helsinki University Central Hospital, Vaasa Central Hospital, Turku University Hospital, Oulu University Hospital, Tampere University Hospital, Päijät-Häme Central Hospital). CONSORT 2010 checklist is shown in Additional file 1.

### Patient evaluation and selection

**Figure 1:**
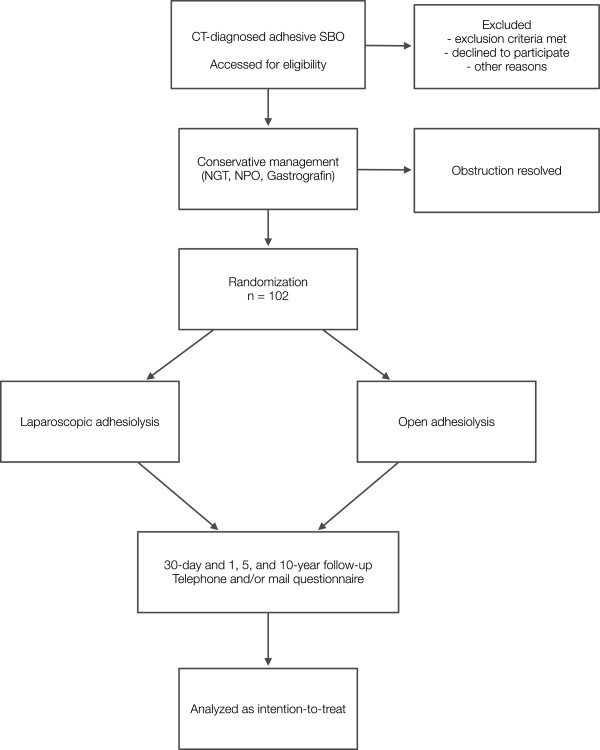
**Flowchart of patients in the trial.** CT - computed tomography, NGT - nasogastric tube, NPO - nil per os, SBO - small bowel obstruction.

Patients with computed tomography-confirmed SBO will be eligible for the study. If no exclusion criteria are present, nasogastric tube is inserted and the patient is admitted to the emergency surgery ward. If the obstruction does not resolve within 12 hours, an oral water-soluble contrast (Gastrografin®) is used. If the contrast has not advanced to the colon within 8 hours and the patient has no signs of spontaneous resolution of obstruction, surgical intervention is considered necessary and the patient is randomized to either open or laparoscopic surgery. The oral water-soluble contrast study has been shown to have 97% sensitivity and 96% specificity in predicting nonoperative resolution of adhesive SBO [5]. Patients can alternatively undergo nonoperative management by fasting and nasogastric tube only if oral water-soluble contrast is contraindicated or not available. If SBO in these patients is not resolved within 48 hours, they are included in the study (Figure 1).

Inclusion criteria:

– All patients with clinical and computed tomography-diagnosed adhesive small bowel obstruction

– Obstruction is not relieved by conservative methods (nasogastric tube, nil per os) including Gastrografin® is not passed to colon within 8 hours (48-hour conservative treatment without Gastrografin® is allowed if Gastrografin® is contraindicated (e.g. allergy) or not available)

Exclusion criteria:

– Strong suspicion of strangulation or clinical peritonitis requiring an urgent operative intervention

– Previously confirmed or strongly suspected peritoneal carcinosis

– Previously confirmed wide diffuse adhesions of the abdominal cavity

– Previous open surgery for endometriosis

– Previous generalized peritonitis (not including local peritonitis such as appendicitis)

– Active abdominal malignancy or remission of less than 10 years’ duration

– Previous radiotherapy of the abdominal region

– Previous obesity surgery

– 3 or more earlier open abdominal operations (not including caesarean section(s))

– Suspicion of other cause for obstruction than adhesions in CT-scan

– Recent abdominal operation (within 30 days)

– Previous laparotomy for aorta or iliac vessels

– Crohn's disease

– Anesthesiological contraindication for laparoscopy

– No informed consent

– Age less than 18 years or over 95 years

– Pregnancy

– Patient living in institutionalized care (such as health centre ward), not including retirement homes

– A hospital stay of more than one week prior to surgical consultation

### Randomization procedure

Patients are randomly allocated (1:1) to either laparoscopic or open surgery. Randomization is done using block randomization with randomly varying block size (2–6) stratified by each study center. Cards with participants’ randomization number and randomization group are sealed within numbered envelopes. Randomization and sealing within envelopes is done at the main research center (Helsinki) and letters are sent to each participating center at the beginning of the trial. The envelope is opened only after patient fulfills inclusion criteria, none of the exclusion criteria are met, and patient has agreed to participate in the study and has given a written consent. Envelopes are opened in numerical order. Operation is scheduled after randomization.

### Intervention

#### Pre- and perioperative treatment

Fluid balance and electrolyte disturbancies are corrected. Prophylactic cefuroxime 1500 mg and metronidazole 500 mg are administered intravenously just before the incision. An epidural catheter may be placed if recommended by the anesthesiologist. A nasogastric tube is inserted.

#### Laparoscopic technique

The first port is inserted using open approach or by using an optic port. Subsequent ports are inserted under direct vision. The location of the ports is left to the surgeons discretion. The abdominal cavity is inspected and the caecum located and identified. Laparoscopic forceps are used to examine the small bowel starting from the terminal ileum until the transition site is identified. Dilated small bowel loops are not grasped, but can be mobilized by grasping the mesenterium. Once the transition site is identified, the obstructing adhesions are divided and the bowel is inspected for vitality. Ports are removed under vision, and possible bleeding is primarily controlled by ligatures. The fascial holes of ports over 5 mm are closed. A nasogastric tube is left in place.

#### Criteria for conversion to open surgery

– Confirmed or suspected small bowel perforation, which is not amenable for laparoscopic suturing

– A transition site is not identified

– The reason for obstruction is not found

– Peritoneal carcinosis is detected

– The presence of widespread diffuse adhesions

– Need for bowel resection - conversion can be made to minilaparotomy to exteriorize the small bowel section requiring resection

#### Open surgical technique

A midline incision is made and the abdominal cavity is inspected. The small bowel is examined until the transition site is located. Adhesions causing obstruction are divided. Excess fluid within small bowel are pushed into the stomach and the stomach is emptied using a nasogastric tube. The fascia is closed using continuous or interrupted sutures at surgeon’s discretion.

#### Postoperative treatment

The nasogastric tube is kept in place until the secretion is less than 500 ml per 8 hours. After the removal of the nasogastric tube, the patient can drink up to 200 ml per 6 hours. If no nausea develops, patient may drink freely. Proton pump inhibitors are used for the length of the hospital stay. Trombosis profylaxis is commenced 6 hours after surgery, if there is no suspicion of postoperative hemorrhage. Ibuprofen, paracetamol, tramadol, and oxycodone can be used for pain. Pain is evaluated using a visual analogue scale daily and before administering pain killers.

#### Criteria for discharge

– Passage of stool

– The patient tolerates per oral nutrition

– Sufficient pain relieve is achieved with ibuprofen, paracetamol, and/or tramadol.

#### Unresolving obstruction after surgery

If the obstruction if not resolved in spite of surgical treatment, the patient can undergo radiological imaging studies and/or surgical exploration (open or laparoscopic) at the discretion of the surgeon.

#### Surgeons

The same surgeons perform both open and laparoscopic operations. All participating surgeons must have solid experience and skills of complex laparoscopic procedures, and need to have perfomed at least two laparoscopic adhesiolysis for small bowel obstruction before operating on patients participating in the trial.

#### Follow-up

Patients of working age are given sick leave. The length of sick leave is at the discretion of treating physician, who is taking into consideration the patient’s age and type of work (physical or desk job). A follow-up call in scheduled within 30 days, and return to work, possible late complications and readmissions are registered. Follow-up questionnaires are sent 1, 5 and 10 years after the randomization, and in case of no response, patients are contacted by telephone. Information about possible hernias and recurrent bowel obstructions is solicited.

### Primary endpoint

– Length of post-operative hospital stay (days)

### Secondary endpoints

– Passage of stool (post-operative days)

– Commencement of enteral nutrition (post-operative days)

– 30-day mortality

– Complications (all causes), graded by Clavien-Dindo classification

– Number of participants with iatrogenic small bowel lesions

– Number of participants with readmission(s)

– Number of participant with failure to resolve obstruction

– Pain scores on the Visual Analog Scale

– Length of epidural catheter analgesia (days)

– Total need of opioids in milligrams

– Length of sick leave (days)

– Conversion rate (laparoscopic group)

### Tertiary endpoints

– Number of participants who develop ventral hernia

– Number of patient with recurrent adhesive small bowel obstruction

### Data collection and analysis

Data will be collected by using an electronical case report form, and statistically analyzed in the main research center (Helsinki) once the trial is completed. Continuous variables will be compared using t-test or Mann–Whitney-U-test. Categorical variables will be compared using Fischer’s exact-test or Chi-square-test. Groups will be analyzed as intention-to-treat. An interim analysis will be made when 52 patients have been randomized and treated.

### Sample size calculation

Based on preliminary retrospective analysis on laparoscopic and open adhesiolysis we have estimated the standard deviation to be 3.75 days in laparoscopic group and 5 days in open surgery group. Sample size is calculated to be able to demostrate 2.5 day difference in the post-operative length of stay. 102 patients are needed to achieve 80% power with a significance level of 0.05.

### Registration

This trial has been registered at ClinicalTrials.gov (Identifier: NCT01867528).

## Discussion

While laparoscopy has become the treatment-of-choice in acute cholecystitis, acute appendicitis, and perforated peptic ulcer, there are still areas of emergency surgery that are under debate [6,7]. It has been suggested that purulent peritonitis caused by acute diverticulitis can be treated by laparoscopic lavage [8], and randomized studies are on their way to either prove or disprove this approach [9,10].

As laparoscopic surgery is becoming more common in emergency surgery, adhesive SBO is the obvious next target for a laparoscopic approach [1]. Although there are several retrospective series, and meta-analyses comparing open approach to laparoscopy, there are no prospective, randomized studies. A search for ongoing trials reveals that, except for this trial, there are no other prospective, randomized trials enrolling patients at the moment of writing. Although previous retrospective series have shown association of less complications and shorter hospital stay with the laparoscopic approach, all previous retrospective series are more or less biased as the easiest cases are selected for laparoscopic approach.

This trial aims to provide level Ib evidence for the use of laparoscopy in the treatment of adhesive SBO that is not resolving by conservative means. Two large meta-analyses have shown that the advancement of oral contrast agent (Gastrografin) to the colon predicts that the obstruction would resolve with 0.97 sensitivity and 0.96 speficity [5,11]. Optimal timing of the abdominal radiograph to predict the success of nonoperative management is unknown [5]. In this study, the sensitivity, specificity, positive and negative likelihood ratios for waiting 4–8 hours were similar to waiting 24 hours before abdominal radiograph is carried out [5,11]. Thus, there appears to be no advantage of waiting more than 4–8 hours. In this trial, if the oral contrast agent is not detected in the colon after 8 hours the conservative management is considered a failure and operative management is warranted. Further, the intestine is decompressed using a nasogastric tube for a minimum of 12 hours before commencing the oral contrast agent study. Thus, in total, the nonoperative management trial takes at minimum 20 hours.

Because there are several exclusion criteria in this trial, the patients are selected, and the results will not be applicable to all patients presenting with an adhesive SBO. These exclusion criteria, however, should not be regarded as absolute contraindications for laparoscopic approach. Many of the exclusion criteria are relative contraindications (peritonitis, suspicion of other cause than adhesions, pregnancy, wide diffuse adhesions, peritoneal carcinosis) or predictors of failure of laparoscopic approach (endometriosis, earlier generalized peritonitis, over 3 open procedures, radiotherapy, vascular procedures) [12]. Furthermore, earlier obesity surgery is an indication for laparoscopic approach, and these patients are excluded because we do not think it is ethical to randomize them for open approach. Some of the exclusion criteria (age >95 years, earlier abdominal surgery within 30 days, patient living in an institutional care, or prior hospital stay of over 1 week) were included to reduce the morbidity of the patients included in the study, as inclusion of a few of these patients in one of the arms would create a strong bias (i.e. the length of stay would be longer because of the morbidity of the patients, not because of the approach used).

The trial has been recruiting since summer 2013. Enrollment of the patients is estimated to last for 4–5 years and primary end-point results are estimated to be available in 2018.

## Abbreviations

SBO: Small bowel obstruction.

## Competing interests

The authors declare that they have no competing interests.

## Authors’ contributions

VS, PM, AL drafted the manuscript. All authors participated in the design of the trial and/or are the main organizing local investigators at the participating hospitals. All authors have read, revised, and approved the final manuscript.

## Pre-publication history

The pre-publication history for this paper can be accessed here:

http://www.biomedcentral.com/1471-2482/14/77/prepub

## Supplementary Material

Additional file 1 CONSORT 2010 checklist of information to include when reporting a randomised trial*. Click here for file
